# Frequency of breast cancer with hereditary risk features in Spain: Analysis from GEICAM “El Álamo III” retrospective study

**DOI:** 10.1371/journal.pone.0184181

**Published:** 2017-10-06

**Authors:** Iván Márquez-Rodas, Marina Pollán, María José Escudero, Amparo Ruiz, Miguel Martín, Ana Santaballa, Purificación Martínez del Prado, Norberto Batista, Raquel Andrés, Antonio Antón, Antonio Llombart, Antonio Fernandez Aramburu, Encarnación Adrover, Sonia González, Miguel Angel Seguí, Lourdes Calvo, José Lizón, Álvaro Rodríguez Lescure, Teresa Ramón y Cajal, Gemma Llort, Carlos Jara, Eva Carrasco, Sara López-Tarruella

**Affiliations:** 1 Servicio de Oncología Médica, Hospital General Universitario Gregorio Marañón, Madrid, Spain; 2 Spanish Breast Cancer Research Group (GEICAM), San Sebastian de los Reyes, Spain; 3 Instituto de Salud Carlos III, Madrid, Spain; 4 Servicio de Oncología Médica, Instituto Valenciano de Oncología, Valencia, Spain; 5 Servicio de Oncología Médica, Hospital Universitari i Politècnic La Fe, Valencia, Spain; 6 Servicio de Oncología Médica, Hospital de Basurto-Osakidetza, Bilbao, Spain; 7 Servicio de Oncología Médica, Hospital Universitario de Canarias, Santa Cruz de Tenerife, Spain; 8 Servicio de Oncología Médica, Hospital Lozano Blesa, Zaragoza, Spain; 9 Servicio de Oncología Médica, Hospital Universitario Miguel Servet, Zaragoza, Spain; 10 Servicio de Oncología Médica, Hospital Arnau de Vilanova, Valencia, Spain; 11 Sección de Oncología Médica, Complejo Universitario de Albacete, Albacete, Spain; 12 Servicio de Oncología Médica, Servicio de Oncología Médica, Hospital Mútua de Terrassa, Barcelona, Spain; 13 Servicio de Oncología Médica, Hospital de Sabadell-Consorcio Sanitario Parc Taulì de Sabadell, Barcelona, Spain; 14 Servicio de Oncología Médica, Complejo Hospitalario Universitario A Coruña, A Coruña, Spain; 15 Servicio de Oncología Médica, Hospital Universitario Sant Joan, Alicante, Spain; 16 Servicio de Oncología Médica, Hospital General Universitario de Elche, Elche, Spain; 17 Servicio de Oncología Médica, Hospital de Sant Pau, Barcelona, Spain; 18 Unitat de Consell Genetic, Institut Oncologic del Valles, Terrasa, Spain; 19 Unidad de Oncología, Hospital Universitario Fundación Alcorcón, Alcorcon, Madrid, Spain; CNR, ITALY

## Abstract

**Purpose:**

To determine the frequency of breast cancer (BC) patients with hereditary risk features in a wide retrospective cohort of patients in Spain.

**Methods:**

a retrospective analysis was conducted from 10,638 BC patients diagnosed between 1998 and 2001 in the GEICAM registry “El Álamo III”, dividing them into four groups according to modified ESMO and SEOM hereditary cancer risk criteria: *Sporadic breast cancer group (R0)*; *Individual risk group (IR)*; *Familial risk group (FR)*; *Individual and familial risk group (IFR)* with both individual and familial risk criteria.

**Results:**

7,641 patients were evaluable. Of them, 2,252 patients (29.5%) had at least one hereditary risk criteria, being subclassified in: FR 1.105 (14.5%), IR 970 (12.7%), IFR 177 (2.3%). There was a higher frequency of newly diagnosed metastatic patients in the IR group (5.1% vs 3.2%, p = 0.02). In contrast, in RO were lower proportion of big tumors (> T2) (43.8% vs 47.4%, p = 0.023), nodal involvement (43.4% vs 48.1%, p = 0.004) and lower histological grades (20.9% G3 for the R0 vs 29.8%) when compared to patients with any risk criteria.

**Conclusions:**

Almost three out of ten BC patients have at least one hereditary risk cancer feature that would warrant further genetic counseling. Patients with hereditary cancer risk seems to be diagnosed with worse prognosis factors.

## Introduction

Breast cancer is the most frequent malignancy in women [1]. In Spain, it is estimated an age-standardized (European standard population) incidence of 85 cases per 100,000 women [2], that would be translated in 25,200 new cases per year. Breast cancer deaths are estimated to be 18% of cancer mortality [3]. Known risk factors are age, late and non-parity, post-menopausal status, and familial background. Preventive and early diagnostic strategies are necessary to reduce the disease burden. However, these strategies vary among countries and even within regions of the same country [4], while there is an increasing interest in adapting screening strategies to the basal breast cancer risk [5]. In this sense, identifying high-risk groups in terms of frequency and prognosis is mandatory for a rationale preventive approach.

It is widely described in the literature that up to 25% of breast cancer patients have a familial/hereditary background, that can be explained through a genetic condition only in a small percentage [6]. Population studies that support this data are scarce, and whether these patients have different prognostic factors or not is a term of debate. Identification of patients at risk of hereditary breast cancer is especially important for those cases that harbor pathological genetic germline mutations in BRCA1 or 2.

Recently, the Spanish Medical Oncology Society (SEOM) have suggested clinical criteria for genetic test selection of hereditary breast cancer patients through a clinical guideline [7]. In the European context, the European Society for Medical Oncology (ESMO) has its own guidelines [6]. In the North American context, the criteria seem to be less restrictive [8].

The implications of genetic testing are nowadays going beyond the prevention and early detection area, influencing also therapeutic decisions with the use of specific treatments, such as oral PARP inhibitors or platinum-based regimens. Currently, several clinical trials are ongoing for different clinical scenarios with these treatments, from the metastatic disease to the adjuvant setting, being in the spotlight of the oncology breast cancer community [9].

In summary, a better understanding of the epidemiological landscape of breast cancer patients with hereditary risk features is of interest.

*El Álamo* Project is a retrospective observational study that includes 26,658 breast cancer patients diagnosed between 1990 and 2001 across 43 Spanish Hospitals and distributed in three cohorts: El Alamo I with 4,532 patients diagnosed between 1990 and 1993, El Alamo II with 10,849 patients diagnosed between 1994 and 1997 and El Alamo III with 11,277 patients diagnosed between 1998 and 2001. El Alamo project has the aim to describe patterns of presentation, management and outcomes of breast cancer in Spain [10, 11]. The latest version, *El Álamo III*, included for the first time the familial background of patients, in addition to clinical and personal features linked to hereditary risk (i.e age, bilaterality, triple negative histology). With more than eleven thousand invasive breast cancer patients diagnosed in 11 of the 17 Spanish regions [12], this is an unique opportunity to explore the previously mentioned questions regarding hereditary breast cancer epidemiology in Spain, since no studies of this kind are currently available in the European context.

The objectives of this study are to analyze the frequency and clinical/pathological characteristics of Spanish invasive breast cancer patients with hereditary risk features.

## Patients and methods

### Compliance with ethical standards

All procedures performed in this study were in accordance with the ethical standards of the participant institutions and with the 1964 Helsinki declaration and its later amendments or comparable ethical standards. “Comité Etico de Investigación Clínica del Area 1” IRB reviewed and approved the Alamo project.

### Study design

This is a retrospective analysis from *El Álamo III* project that included 11,277 breast cancer patients. *El* Alamo focused on female breast cancer and only some centers recruited a small number of male patients (37 cases, 0.3% of the total sample), so they were excluded from this analysis. Non invasive carcinoma breast cancer cases (602 patients, 5.3%) were also excluded.

Questionnaires including data regarding individual tumor information and familial features were completed by the clinical investigators (it can be found in reference [12]. Based on these data, hereditary risk groups were defined, according to the following modified SEOM and ESMO criteria [6, 7].

### Modified ESMO-SEOM Criteria for hereditary breast cancer risk

The individual criteria were: breast cancer diagnosis under 40 years or breast cancer diagnosis under 50 years if one of the following: triple negative breast cancer (TNBC) histology and/or bilateral (synchronous or metachronous) breast cancer or breast cancer at any age together with ovarian cancer. The familial criteria were: the presence of first or second degree relatives with the following features: 3 relatives (including the patient) with breast and /or ovarian cancer or 2 relatives (including the patient), if the relative fulfill any of the individual criteria above mentioned, regardless degree; or 2 relatives (including the patient) if is first degree and diagnosed with breast and/or ovarian cancer.

According to the individual and familial criteria, patients were divided into 4 different subgroups: *Sporadic breast cancer group (R0)* (Control group) without individual or familial risk criteria; *Individual risk group (IR)* with no familial or not determined (ND) familial risk, but with individual risk criteria; *Familial risk group (FR)* with no individual or ND individual risk, but with familial risk criteria; *Individual and familial risk group (IFR)* with both individual and familial risk criteria. Global hereditary risk group (GHR) comprises the three last categories, namely IR or FR or IFR.

### Statistical methods

Chi-square and unpaired t student/Anova were used to compare categorical and continuous variables respectively. All statistical tests had a significance level of 0.05 unless stated otherwise. Data were analysed using SPSS^®^ version 21 (IBM corporation).

## Results

### Frequency and characteristics of hereditary risk breast cancer patients

From 1998 to 2001, 10,638 women with invasive breast cancer were included in the study. Patients who had enough information to be sub-classified as one of the four risk subgroups accounted for 7,641 (71.8%). The individual and familial risk criteria for hereditary breast cancer of the global sample are described in Table 1. Eleven out of the 17 different Spanish regions were represented (64.7%).

**Table 1:** Individual and familial features distribution in the global sample.

Individual risk features: 1902 patients (17,9%) had not information		
	N	%
Age < 40 y^a^	973	11,1
> = 40 y & <50y & TNBC	122	1,4
> = 40 y & <50y & Bilateral	19	0,2
Ovarian cancer	33	0,4
Non personal risk features	7589	86,9
Total	8736	100,0
Familial background features: 1944 patients (18,3%) had not information		
	N	%
2 relatives (patient + relative with ovarian cancer)	111	1,3
3 or more relatives (patient + 2 relatives with BC and/or ovarian cancer, regardless degree)	434	5,0
2 relatives (patient + 1 BC of first degree)	737	8,5
No family features^b^	7412	85,2
Total	8694	100,0

^a^ 67 patients were also TNBC; 6 were also bilateral BC; 4 had also ovarian cancer; 1 was TNBC and bilateral BC;

^b^528 of them had 1 relative but in second degree with BC, not considered in consequence at hereditary risk

Of these evaluable patients, 2,252 patients (29.5%) had at least one hereditary risk criteria, constituting the global hereditary risk group (GHR). The 5,389 (70.5%) remaining patients, with no risk features, were considered the R0 group (Table 2). Table 3 describes the pathological characteristics of patients evaluable for hereditary risk (N = 7,641).

**Table 2:** Hereditary risk distribution.

2533 patients (23,8%) had some feature missing	Excluding those without information	
	N	%
R0 (Sporadic)	5389	70,5
IFR (both individual and familial)	177	2,3
IR (only individual risk)	970	12,7
FR (only familial risk)	1105	14,5
Total	7641	100,0

**Table 3:** Description of the TNM, histological subtype and grade of the evaluable patients, including TN patients. Only pathological T and N were considered.

	R0	GHR (IFR+IR+FR)	p-value	IFR	IR	FR	p-value
	N (%)	N (%)		N (%)	N (%)	N (%)	
pT (N = 6746)	N = 4813	N = 1933	0.023 (T0+T1 vs T2+T3+T4)	N = 153	N = 804	N = 976	<0.001 (T0+T1 vs T2+T3+T4)
T0 (Tis)	10 (0.2)	9 (0.5)		0	2 (0.2)	7 (0.7)	
T1 (52 T1mic)	2703 (56.2)	1017 (52.6)		87 (56.8)	365 (45.5)	565 (57.9)	
T2	1738 (36.1)	752 (38.9)		57 (37.3)	361 (44.9)	334 (34.2)	
T3	164 (3.4)	74 (3.8)		6 (3.9)	46 (5.7)	22 (2.3)	
T4	180 (3.7)	65 (3.4)		2 (1.3)	21 (2.6)	42 (4.3)	
TX	18 (0.4)	16 (0.8)		1 (0.7)	9 (1.1)	6 (0.6)	
pN (N = 6746)	N = 4813	N = 1933	0.004 (NX not analysed)	N = 153	N = 804	N = 976	<0.001 (NX not analysed)
N0	2721 (56.6)	1003 (51.9)		84 (54.9)	385 (47.9)	534 (54.7)	
N1	1671 (34.7)	748 (38.7)		55 (35.9)	347 (43.2)	346 (35.5)	
N2	243 (5.0)	112 (5.8)		7 (4.6)	55 (6.8)	50 (5.1)	
N3	76 (1.6)	28 (1.4)		5 (3.3)	9 (1.1)	14 (1.4)	
NX	102 (2.1)	42 (2.2)		2 (1.3)	8 (1.0)	32 (3.3)	
M (N = 7641)	N = 5389	N = 2252	0.256 (M ND not analysed)	N = 177	N = 970	N = 1105	0.02 (M ND not analysed)
M0	5219 (96.8)	2165 (96.1)		172 (97.2)	920 (94.9)	1073 (97.1)	
M1	167 (3.1)	81 (3.6)		5 (2.8)	47 (4.8)	29 (2.6)	
M ND	3 (0.1)	6 (0.3)		0	3 (0.3)	3 (0.3)	
Grade H (N = 7641)	N = 5389	N = 2252	<0.001 (GX not analysed)	N = 177	N = 970	N = 1105	<0.001 (GX not analysed)
GX	947 (17.6)	429 (19.0)		30 (16.9)	188 (19.4)	211 (19.1)	
G1	1140 (21.2)	335 (14.9)		21 (11.9)	97 (10.0)	217 (19.6)	
G2	2177 (40.3)	817 (36.3)		54 (30.5)	349 (36.0)	414 (37.5)	
G3	1125 (20.9)	671 (29.8)		72 (40.7)	336 (34.6)	263 (23.8)	
Subtypes (N = 7641)	N = 5389	N = 2252	<0.001(unknown not analysed)	N = 177	N = 970	N = 1105	<0.001 (unknown not analysed)
TN	208 (3.9)	225 (10.0)		33 (18.6)	159 (16.4)	33 (3.0)	
Her2+	552 (10.2)	224 (9.9)		15 (8.5)	124 (12.8)	85 (7.7)	
RH+ Her2-	1452 (26.9)	499 (22.2)		36 (20.3)	200 (20.6)	263 (23.8)	
Unknown	3177 (59.0)	1304 (57.9)		93 (52.6)	487 (50.2)	724 (65.5)	

### Analysis of prognostic factors in sporadic and hereditary breast cancer groups

In the univariate analysis we found that R0 group presented a lower proportion of big tumors (≥ T2) than the GHR group (43.8% vs 47.4%, p = 0.023), a lower proportion of nodal involvement (43.4% vs 48.1%, p = 0.004) and lower histological grades (20.9% G3 for the R0 vs 29.8% for the GHR group, p<0.001). Metastases at diagnosis were present in similar proportion in both groups (3.2% vs 3.9%, p = 0.26). As expected, a higher proportion of TNBC was found in the GHR group, given that the TN phenotype is included in the criteria to define hereditary cancer (Table 3). In order to rule out an effect by TN phenotype itself in this observation, we conducted the same analysis excluding from all subgroups the TN patients (Table 4), and we found that RO maintained a statistically significant lower proportion of nodal involvement (43.6% vs 48.7%, p = 0.00173) and lower histological grades (19.4% G3 for the R0 vs 26.8% for the GHR group, p<0.001). However, tumor size was not statistically significant between the two subgroups (Table 4)

**Table 4:** Description of the TNM, histological subtype and grade of the evaluable patients, excluding those TN patients. Only pathological T and N were considered.

	R0	GHR (IFR+IR+FR)	p-value	IFR	IR	FR	p-value
	N (%)	N (%)	0.061 (T0+T1 vs T2+T3+T4)	N (%)	N (%)	N (%)	
**pT (N = 6382)**	**N = 4637**	**N = 1745**		**N = 125**	**N = 672**	**N = 948**	0.146 (T0+T1 vs T2+T3+T4)
T0 (Tis)	10 (0.2)	9 (0.5)		0	2 (0.3)	7 (0.7)	
T1 (49 T1mic)	2634 (56.8)	936 (53.6)		68 (54.4)	309 (46.0)	559 (59.0)	
T2	1640 (35.4)	658 (37.7)		48 (38.4)	292 (43.4)	318 (33.6)	
T3	158 (3.4)	66 (3.8)		6 (4.8)	40 (6.0)	20 (2.1)	
T4	177 (3.8)	61 (3.5)		2 (1.6)	20 (3.0)	39 (4.1)	
TX	18 (0.4)	15 (0.9)		1 (0.8)	9 (1.3)	5 (0.5)	
**pN (N = 6382)**	**N = 4637**	**N = 1745**	0.00173 (NX not anlysed)	**N = 125**	**N = 672**	**N = 948**	0.001 (NX not anlysed)
N0	2614 (56.4)	896 (51.3)		67 (53.6)	307 (45.7)	522 (55.0)	
N1	1617 (34.9)	678 (38.9)		46 (36.8)	299 (44.5)	333 (35.1)	
N2	229 (4.9)	106 (6.1)		6 (4.8)	51 (7.6)	49 (5.2)	
N3	76 (1.6)	24 (1.4)		4 (3.2)	7 (1.0)	13 (1.4)	
NX	101 (2.2)	41 (2.3)		2 (1.6)	8 (1.2)	31 (3.3)	
**M (N = 7208)**	**N = 5181**	**N = 2027**	0.1139 (M ND not analysed)	**N = 144**	**N = 811**	**N = 1072**	0.01383 (M ND not analysed)
M0	5020 (96.9)	1945 (96.0)		139 (96.5)	766 (94.5)	1040 (97.0)	
M1	159 (3.1)	77 (3.8)		5 (3.5)	43 (5.3)	29 (2.7)	
M ND	2 (0.0)	5 (0.2)		0	2 (0.2)	3 (0.3)	
**Grade H (N = 7208)**	**N = 5181**	**N = 2027**	<0.001 (GX not analysed)	**N = 144**	**N = 811**	**N = 1072**	<0.001 (GX not analysed)
GX	912 (17.6)	399 (19.7)		26 (18.1)	168 (20.7)	205 (19.1)	
G1	1128 (21.8)	324 (16.0)		19 (13.2)	88 (10.9)	217 (20.2)	
G2	2134 (41.2)	760 (37.5)		49 (34.0)	303 (37.3)	408 (38.1)	
G3	1007 (19.4)	544 (26.8)		50 (34.7)	252 (31.1)	242 (22.6)	
**Subtypes (N = 7208)**	**N = 5181**	**N = 2027**	0.079 (unknown not analysed)	**N = 144**	**N = 811**	**N = 1072**	<0.001 (unknown not analysed)
Her2+	552 (10.7)	224 (11.1)		15 (10.4)	124 (15.3)	85 (7.9)	
RH+ Her2-	1452 (28.0)	499 (24.6)		36 (25.0)	200 (24.7)	263 (24.5)	
Unknown	3177 (61.3)	1304 (64.3)		93 (64.6)	487 (60.0)	724 (67.6)	

### Analysis according to different hereditary risk subgroups

Comparing each specific GHR subtype with sporadic cases, we observed that the differences seen before are only observed for the IR group. In contrast, similar clinic-pathological features were seen between R0 and IFR and FR groups respectively (Table 3). Moreover, there was a higher frequency of newly diagnosed metastatic patients in the IR group (5.1% vs 3.2%, p = 0.02)

## Discussion

According to this large and representative sample of the Spanish breast cancer landscape, we can say that three out of ten patients have, at least, one hereditary breast cancer risk feature, and, in consequence, could be candidate for genetic testing and counselling. Overall, patients with hereditary cancer risk features have larger tumors and more frequently nodal involvement in comparison to patients without hereditary cancer risk features, while both subgroups have a similar rate of distant metastases at initial diagnosis. However, these differences probably are related to the greater aggressiveness observed in patients fulfilling the individual criteria. Interestingly, when patients with TNBC were excluded for this analysis, presence of nodal involvement and higher grades, although not tumor size, remained higher in patients with hereditary risk features.

Strong points of this study are the large number of patients analyzed and the representativeness’ of Spanish population, since two thirds of the regions are represented. Few studies exist in Spain analyzing the frequency of different familial cancer from a population point of view, with the exception of melanoma [13], pancreatic [14] and colorectal cancer [15]. However, several limitations must be also taken into account when interpreting our results. First, the retrospective nature of our work that could concur in some bias, since almost 30% of patients analyzed lack information to be included in a given risk group. Based on that, we decided to analyze only those that could be categorized in a risk group. This could be a selection bias, over-estimating the risk percentage. However, due to the large number of patients analyzed, this possibility might be ameliorated.

Another weakness, in order to classify patients in a given risk group, is the fact that nearly 60% of patients had an unknown HER2 status, in consequence, a substantial number of patients, could not be evaluated regarding the TN phenotype, one of the major risk factors for hereditary breast cancer. Within the time-frame of data collection (patients diagnosed from 1998 to 2001), although the role of HER2 was well known as a prognostic factor, the determination of this biomarker was not widely used, given that appropriate targeted therapy was only available for metastatic patients.

Patients with hereditary cancer risk features have worse pathological risk factors, according to T and N status, and to histological grade, all well-known bad prognosis factors. Data of prognosis from patients with known BRCA 1 and 2 mutations are conflicting in literature. A recent meta-analysis did not detect differences in breast cancer specific survival rate in BRCA2 mutation carriers when compared to sporadic ones [16]. I contrast, another meta-analysis confers a poorer prognosis for patients with BRCA1 mutations [17]. Another recent meta-analysis confer worse overall survival to BRCA1 mutation carriers and worse breast cancer specific survival [18]. In our study, we did not analyze survival, and we did not have data regarding BRCA1 and 2 status, so in consequence we cannot put our data into the context of theses meta-analysis.

One could think that patients concerned with their familial background are more prone to intensive surveillance, both by themselves and by their health care givers, what should be translated into earlier diagnostic presentations, something that is not reflected by our data. This is true in other familial cancers, such as melanoma, where patients at familial risk in Spain present with better prognosis pathological factors [13]. However, since the subgroup responsible for these differences is the individual risk group, which is enriched with the triple negative phenotype, the known biological aggressiveness of this subtype may account for these differences in TNM presentation.

It is important to analyze if our data are comparable to other countries. Our results are according to what is described in general literature [6]. However, studies conducted in other countries searching for similar endpoints as our present work, revealed mixed results. In a British study with more than 5,000 BC patients, a positive family history of BC (with no more specific details) was found in 22.2%, in contrast to 16.8% (14.5% FR and 2.3% IFR groups) found in our work [19]. In this study, a younger age of presentation was found among patients with family history. In a pooled analysis with more than 47,000 BC patients, in which 92% were of European ancestry, revealed that 11% of patients were <40y, 20% had a positive first degree family history and that 14% were TNBC. 18% of patients with TNBC had also a positive family history of cancer [20]. In African-American women, a study found that 16% of BC patients had first degree family history, 3% ovarian cancer and 15% were TNBC [21]. Finally, in Chinese population, a lower proportion of BC with family history (5.1%) was described in a study focused in Han Chinese population, the majority of Chinese population ethnicity [22]. These results reveal that family history and other risk factors associated with increased hereditary risk could be dependent of geographical origin, although the limitations of the heterogeneity of the different studies should be taken into account.

Finally, the practical consequences of our findings should be taken into consideration. In general, it is estimated that, according to different institutional series in Spain and western countries, BRCA 1 or 2 mutations are present from 7% to 20% of selected and unselected patients in western countries [23–27]. With this in mind, and since genetic testing will be easier and cheaper in the near future, our findings suggests that it is urgently needed an increase in efforts to facilitate the detection and proper management of patients and relatives harboring genetic mutations and/or high familial risk features.

## References

[1] Ferlay J, Soerjomataram I, Dikshit R, Eser S, Mathers C, Rebelo M et al (2015) Cancer incidence and mortality worldwide: Sources, methods and major patterns in GLOBOCAN 2012. Int J Cancer 136:E359–E386. doi: 10.1002/ijc.29210 25220842 10.1002/ijc.29210

[2] Ferlay J, Steliarova-Foucher E, Lortet-Tieulent J, Rosso S, Coebergh JWW, et al (2013) Cancer incidence and mortality patterns in Europe: Estimates for 40 countries in 2012. Eur J Cancer 49:1374–1403. doi: 10.1016/j.ejca.2012.12.027 23485231 10.1016/j.ejca.2012.12.027

[3] López-Abente G, Aragonés N, Pérez-Gómez B, Pollán M, García-Pérez J, Ramis R, Fernández-Navarro P (2014) Time trends in municipal distribution patterns of cancer mortality in Spain. BMC Cancer 14:535 doi: 10.1186/1471-2407-14-535 25060700 10.1186/1471-2407-14-535 PMC4124154

[4] Depypere H, Desreux J, Pérez-López FR, Ceausu I, Erel CT, Lambrinoudaki I, et al (2014) EMAS position statement: individualized breast cancer screening versus population-based mammography screening programmes. Maturitas 79:481–486. doi: 10.1016/j.maturitas.2014.09.002 25277123 10.1016/j.maturitas.2014.09.002

[5] Schousboe JT, Kerlikowske K, Loh A, Cummings SR (2011) Personalizing mammography by breast density and other risk factors for breast cancer: analysis of health benefits and cost-effectiveness. Ann Intern Med 155:10–20. doi: 10.7326/0003-4819-155-1-201107050-00003 21727289 10.7326/0003-4819-155-1-201107050-00003 PMC3759993

[6] Paluch-Shimon S, Cardoso F, Sessa C, Balmana J, Cardoso MJ, Gilbert F et al (2016) Prevention and screening in BRCA mutation carriers and other breast/ovarian hereditary cancer syndromes: ESMO Clinical Practice Guidelines for cancer prevention and screening. Ann Oncol Off J Eur Soc Med Oncol 27:v103–v110. doi: 10.1093/annonc/mdw327 27664246 10.1093/annonc/mdw327

[7] Llort G, Chirivella I, Morales R, Serrano R, Sanchez AB, Teulé A et al (2015) SEOM clinical guidelines in Hereditary Breast and ovarian cancer. Clin Transl Oncol Off Publ Fed Span Oncol Soc Natl Cancer Inst Mex 17:956–961. doi: 10.1007/s12094-015-1435-3 26669313 10.1007/s12094-015-1435-3 PMC4689749

[8] Stuckey A, Febbraro T, Laprise J, Wilbur JS, Lopes V, Robison K (2016) Adherence Patterns to National Comprehensive Cancer Network Guidelines for Referral of Women With Breast Cancer to Genetics Professionals. Am J Clin Oncol 39:363–367. doi: 10.1097/COC.0000000000000073 24710121 10.1097/COC.0000000000000073

[9] Sonnenblick A, de Azambuja E, Azim HA, Piccart M (2015) An update on PARP inhibitors—moving to the adjuvant setting. Nat Rev Clin Oncol 12:27–41. doi: 10.1038/nrclinonc.2014.163 25286972 10.1038/nrclinonc.2014.163

[10] Martín M, Mahillo E, Llombart-Cussac A, Lluch A, Munarriz B, Pastor M et al (2006) The «El Álamo» project (1990–1997): two consecutive hospital-based studies of breast cancer outcomes in Spain. Clin Transl Oncol 8:508–518. doi: 10.1007/s12094-006-0051-7 16870541 10.1007/s12094-006-0051-7

[11] C. Jara-Sanchez, A. Ruiz, M. Martin, P. Martínez del Prado, A. Santaballa, A. Llombart-Cussac, et al (2006)J Spanish Breast Cancer Research Group (GEICAM) hospital-based study on breast cancer outcomes: El Álamo project (1990–2001). J Clin Oncol 28, no. 15_suppl

[12] http://www.geicam.org/wp-content/uploads/2017/04/Lib_El_AlamoIII_Anexo_I.pdf.

[13] Márquez-Rodas I, Martín González M, Nagore E, Gómez-Fernández C, Avilés-Izquierdo JA, Maldonado-Seral C et al (2015) Frequency and characteristics of familial melanoma in Spain: the FAM-GEM-1 Study. PloS One 10:e0124239 doi: 10.1371/journal.pone.0124239 25874698 10.1371/journal.pone.0124239 PMC4395344

[14] Mocci E, Guillen-Ponce C, Earl J, Marquez M, Solera J, Salazar-López M-T et al (2015) PanGen-Fam: Spanish registry of hereditary pancreatic cancer. Eur J Cancer Oxf Engl 1990 51:1911–1917. doi: 10.1016/j.ejca.2015.07.004 26212471 10.1016/j.ejca.2015.07.004

[15] Castellví-Bel S, Ruiz-Ponte C, Fernández-Rozadilla C, Abulí A, Muñoz J, Bessa X et al (2012) Seeking genetic susceptibility variants for colorectal cancer: the EPICOLON consortium experience. Mutagenesis 27:153–159. doi: 10.1093/mutage/ger047 22294762 10.1093/mutage/ger047

[16] Shao J, Yang J, Wang J, Qiao L, Fan W, Gao Q et al (2015) Effect of BRCA2 mutation on familial breast cancer survival: A systematic review and meta-analysis. J Huazhong Univ Sci Technol Med Sci Hua Zhong Ke Ji Xue Xue Bao Yi Xue Ying Wen Ban Huazhong Keji Daxue Xuebao Yixue Yingdewen Ban 35:629–634. doi: 10.1007/s11596-015-1481-7 26489613 10.1007/s11596-015-1481-7

[17] Lee E-H, Park SK, Park B, Kim S-W, Lee MH, Ahn SH et al (2010) Effect of BRCA1/2 mutation on short-term and long-term breast cancer survival: a systematic review and meta-analysis. Breast Cancer Res Treat 122:11–25. doi: 10.1007/s10549-010-0859-2 20376556 10.1007/s10549-010-0859-2

[18] Baretta Z, Mocellin S, Goldin E, Olopade OI, Huo D (2016) Effect of BRCA germline mutations on breast cancer prognosis: A systematic review and meta-analysis. Medicine (Baltimore) 95:e4975 27749552 10.1097/MD.0000000000004975 PMC5059054

[19] Melvin JC, Wulaningsih W, Hana Z, Purushotham AD, Pinder SE, Fentiman I et al (2016) Family history of breast cancer and its association with disease severity and mortality. Cancer Med 5:942–949. doi: 10.1002/cam4.648 26799372 10.1002/cam4.648 PMC4864823

[20] Yang XR, Chang-Claude J, Goode EL, Couch FJ, Nevanlinna H, Milne RL et al (2011) Associations of breast cancer risk factors with tumor subtypes: a pooled analysis from the Breast Cancer Association Consortium studies. J Natl Cancer Inst 103:250–263. doi: 10.1093/jnci/djq526 21191117 10.1093/jnci/djq526 PMC3107570

[21] Bethea TN, Rosenberg L, Castro-Webb N, Lunetta KL, Sucheston-Campbell LE, Ruiz-Narváez EA et al (2016) Family history of cancer in relation to breast cancer subtypes in African American women. Cancer Epidemiol Biomark Prev Publ Am Assoc Cancer Res Cosponsored Am Soc Prev Oncol 25:366–373. doi: 10.1158/1055-9965.EPI-15-1068 26721669 10.1158/1055-9965.EPI-15-1068 PMC4767636

[22] Zhou W, Pan H, Liang M, Xia K, Liang X, Xue J et al (2013) Family history and risk of ductal carcinoma in situ and triple negative breast cancer in a Han Chinese population: a case–control study. World J Surg Oncol 11:248 doi: 10.1186/1477-7819-11-248 24083544 10.1186/1477-7819-11-248 PMC3850692

[23] Zugazagoitia J, Pérez-Segura P, Manzano A, Blanco I, Vega A, Custodio A (2014) Limited family structure and triple-negative breast cancer (TNBC) subtype as predictors of BRCA mutations in a genetic counseling cohort of early-onset sporadic breast cancers. Breast Cancer Res Treat 148:415–421. doi: 10.1007/s10549-014-3167-4 25342642 10.1007/s10549-014-3167-4

[24] González-Rivera M, Lobo M, López-Tarruella S, Jerez Y, del Monte-Millán M, Massarrah T et al (2016) Frequency of germline DNA genetic findings in an unselected prospective cohort of triple-negative breast cancer patients participating in a platinum-based neoadjuvant chemotherapy trial. Breast Cancer Res Treat 156:507–515. doi: 10.1007/s10549-016-3792-1 27083178 10.1007/s10549-016-3792-1

[25] Loman N, Johannsson O, Kristoffersson U, Olsson H, Borg A (2001) Family history of breast and ovarian cancers and BRCA1 and BRCA2 mutations in a population-based series of early-onset breast cancer. J Natl Cancer Inst 93:1215–1223. 11504767 10.1093/jnci/93.16.1215

[26] de Sanjosé S, Léoné M, Bérez V, Izquierdo A, Font R, Brunet JM et al (2003) Prevalence of BRCA1 and BRCA2 germline mutations in young breast cancer patients: a population-based study. Int J Cancer 106:588–593. doi: 10.1002/ijc.11271 12845657 10.1002/ijc.11271

[27] Andrés R, Pajares I, Balmaña J, Llort G, Ramón Y Cajal T, Chirivella I et al (2014) Association of BRCA1 germline mutations in young onset triple-negative breast cancer (TNBC). Clin Transl Oncol Off Publ Fed Span Oncol Soc Natl Cancer Inst Mex 16:280–284. doi: 10.1007/s12094-013-1070-9 23982851 10.1007/s12094-013-1070-9

